# Enhanced Fracture Energy and Toughness of UV-Curable Resin Using Flax Fiber Composite Laminates

**DOI:** 10.3390/biomimetics11010071

**Published:** 2026-01-15

**Authors:** Mingwen Ou, Huan Li, Dequan Tan, Yizhen Peng, Hao Zhong, Linmei Wu, Wubin Shan

**Affiliations:** 1 Department of Automotive Engineering, Hunan Electrical College of Technology, Xiangtan 411101, China; 2 The School of Environmental Art and Architecture, Changsha Environmental Protection Vocational College, Changsha 410004, China; 3 School of Intelligent Equipment and Manufacturing, Hunan Electrical College of Technology, Xiangtan 411101, China; 4 Department of Mechanical Engineering, University of Western Australia, Perth, WA 6009, Australia; 5 College of Mechanical and Vehicle Engineering, Hunan University, Changsha 410082, China

**Keywords:** UV-curable resin, flax fiber, composite material, biomimetic structure, mechanical performance, DIC analysis

## Abstract

Ultraviolet (UV) curable resins are widely used in photopolymerization-based 3D printing due to their rapid curing and compatibility with high-resolution processes. However, their brittleness and limited mechanical performance restrict their applicability, particularly in impact-resistant high-performance 3D-printed structures. Inspired by the mantis shrimp’s exceptional energy absorption and impact resistance, attributed to its helicoidal fiber architecture, we developed a Bouligand flax fiber-reinforced composite laminate. By constructing biomimetic helicoidal composites based on Bouligand arrangements, the mechanical performance of flax fiber-reinforced UV-curable resin was systematically investigated. The influence of flax fiber orientation was assessed using mechanical testing combined with the digital image correlation (DIC) method. The results demonstrate that a 45° interlayer angle of flax fiber significantly enhanced the fracture energy of the resin from 1.67 KJ/m^2^ to 15.41 KJ/m^2^, an increase of ~823%. Moreover, the flax fiber-reinforced helicoidal structure markedly improved the ultimate tensile strength of the resin, with the 90° interlayer angle of flax fiber exhibiting the greatest enhancement, increasing from 5.32 MPa to 19.45 MPa.

## 1. Introduction

UV-curable resins are widely used in molding and 3D printing due to their excellent photopolymerization performance, high resolution, and rapid curing capabilities [1,2,3,4,5]. However, their brittleness and low toughness severely limit their applications in fields requiring high mechanical performance (e.g., load-bearing components and impact-resistant). To overcome these limitations, various strategies, including molecular modification of acrylates and the incorporation of nanoparticles, have been proposed. The mechanical performance (e.g., flexural strength, impact strength, and toughness) of UV-curable resins can be improved [6,7,8,9,10]. While these approaches enhance resin performance, challenges remain in achieving both high tensile strength and fracture tolerance simultaneously.

Plant fibers, such as flax fibers, act as a renewable and mechanically robust alternative for reinforcement [11,12,13]. Flax fibers show high tensile strength, stiffness, and energy absorption capability, making them ideal candidates for improving the mechanical performance of polymer composites [14,15,16]. Equally importantly, the hierarchical and helicoidal structures observed in natural materials provide valuable inspiration for designing tough, fracture-tolerant composites [17,18,19,20,21]. The Bouligand structure is a typical helicoidal layered arrangement observed in organisms [22,23,24,25,26]. For example, the dactyl club of the mantis shrimp can deliver high-speed impacts sufficient to fracture hard shells [27,28]. This capability is attributed to its Bouligand-type helicoidal fiber architecture [29]. This coupled structure not only provides high strength and toughness but also distributes stress, absorbs energy, and maintains structural integrity after repeated impacts [22,28,29]. However, the fracture toughness of a single Bouligand structure remains suboptimal. Incorporating plant fibers into this architecture is expected to further enhance both tensile strength and fracture toughness, offering a promising strategy for designing high-performance, impact-resistant composites.

Herein, we developed a biomimetic flax fiber-reinforced UV-curable resin composite with a helicoidal (Bouligand) fiber arrangement fabricated via photopolymerization 3D printing. By varying the interlayer orientation of flax fibers, we investigate their effect on fracture energy, tensile strength, and crack propagation. Mechanical testing and DIC analysis indicate that the flax fiber at a 45° angle significantly increases the fracture toughness of UV-curable resin. Furthermore, the helical structure with flax fiber reinforcement improves the ultimate tensile strength, with the 90° interlayer angle showing an increase from 5.32 MPa to 19.45 MPa. Therefore, we provided a strategy for enhancing the mechanical performance of 3D-printed UV-curable resins and demonstrated how biomimetic design principles can be translated into high-performance polymer composites.

## 2. Experiment Section

### 2.1. Structural Design and Sample Fabrication

**Figure 1:**
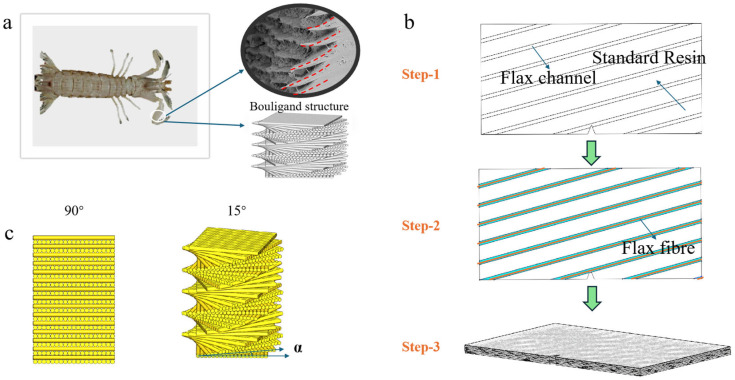
Biomimetic helical structure-based composite material. (**a**) Schematic diagram and biological structure of a mantis shrimp. Adapted from reference [31]. (**b**) Sample fabrication steps of flax fiber/standard resin composite material samples. (**c**) Schematic diagram of a biomimetic helical structure composite material.

The biological structure of the mantis shrimp, specifically its dactyl club, serves as the primary inspiration for this work. The dactyl club is a highly specialized appendage used to strike prey with remarkable force and speed. It can accelerate to speeds of up to 23 m·s^−1^ and deliver high-energy impacts, capable of breaking hard shells [27,28]. The club’s performance is attributed to its unique Bouligand structural composition, which includes a helicoidal arrangement of chitin fibers. This helicoidal structure effectively dissipates stress and absorbs energy, preventing catastrophic failure under repeated impacts. The structure is organized into several functional regions, each contributing to its superior toughness and impact resistance. The outer region consists of highly mineralized chitin fibers, providing hardness, while the inner region features a more complex helicoidal arrangement that offers flexibility and toughness [30]. Figure 1a shows the intricate biological design, showcasing multiple layers and their orientations. The preparation of the biomimetic composite was carried out using the composite material stacking method, with the schematic illustrating the helical laminate preparation steps shown in Figure 1b. For the helical structure composite, channels for flax fibers were pre-designed within the structural layers. The flax fibers were then deployed according to these channels, which were rotated as schematically shown in Figure 1c. The orientation of each adjacent flax fiber layer was incrementally rotated counterclockwise, with rotation angles sequentially set to 15°, 30°, 45°, 60°, 75°, and 90°. This step ensures the creation of a bio-inspired helical structure, enhancing the mechanical performance of the UV-curable resin composite.

All samples were designed using SolidWorks 2020 and fabricated with a photopolymerization 3D printer (Sonic Mini 8K, Phrozen, Shenzhen, China) using 405 nm UV light. The 3D printer features a resolution of 22 µm pixel size and 1152 ppi in the X and Y directions. A commercial standard resin (Anycubic, Shenzhen, China) was used for the rigid matrix, and flax fiber (Guangdong, China) was used as the toughening agent. The SLDASM files were saved as STL files and processed with CHITUBOX (Basic) software to generate the G-code. The G-code was uploaded to the 3D printer for fabrication, with each layer printed at a fixed thickness of 50 µm. The curing time for each layer was set to 10 s, while the curing time of the bottom layer was 30 s. After every six layers of standard UV-curable resin, a layer of flax fiber was manually embedded into the structure. The orientation of each subsequent flax fiber layer was incrementally rotated by a specific angle relative to the previous one, thereby forming a helicoidal fiber architecture. This gradually rotating fiber arrangement mimics the natural Bouligand structure. The specimens measured 60 mm in length, 30 mm in width, and 2 mm in thickness, with both the notch depth and width set to 5 mm. Each specimen was tested three times, and the mechanical performance was reported based on the calculated mean values and standard deviations.

Using a controlled variable approach, we examined how flax fiber orientation affects resin toughness across different layup angles. This method allows clear analysis of how local fiber orientation affects the fracture toughness and tensile strength of UV-curable standard resin. The flax fibers and standard resin composites were fabricated using photopolymerization 3D printing and subjected to mechanical testing. It is noticed that the commercially available UV-curable standard resin (Anycubic Basic UV Resin) was used for 3D printing. Flax fiber, a natural fiber with excellent mechanical properties, is used to enhance the toughness. Embedding fibers in a thick polymer matrix ensures effective load transfer and realistic interaction with the resin. By incrementally rotating the fiber layers (15–90°), a helicoidal architecture is gradually formed, and the observed single-layer effects can be extrapolated to predict the performance of the complete Bouligand-inspired laminate. Six helical structures with different flax fiber orientations (15°, 30°, 45°, 60°, 75°, and 90°) were printed, as shown in Table 1.

### 2.2. Characterization

Mechanical characterization tests were conducted using a universal testing machine (Huaheng, Changsha, China) under vertical tensile loads to evaluate their mechanical properties. By analyzing the tensile curves and the morphology of the fracture surfaces, a series of parameters such as maximum force, fracture energy, work of fracture, and ultimate tensile strength were obtained. The samples were stretched to failure at a displacement-controlled speed of 1 mm/min, using a calibrated load cell for force measurement. The stress and strain were utilized to describe the mechanical behavior of the samples. Stress was defined as the applied force divided by the initial cross-sectional area of the samples, while strain was defined as the measured displacement divided by the initial gauge length. In addition, the modulus was defined as the slope of the stress–strain curve within the initial linear elastic region (ε = 0−0.025). Failure strain was taken as the maximum strain observed from the stress–strain curve. The trapezoidal rule was employed to calculate the toughness, determining the area under the stress–strain curve up to the point of material failure. In addition, the same samples (60 mm in length, 30 mm in width, and 2 mm in thickness, with both the notch depth and width set to 5 mm) as described above underwent testing via the 2D DIC method. Crack propagation was analyzed both from the force–displacement response and via 2D DIC strain mapping, which allowed visualization of strain concentration and fracture process zones. DIC measurements and analyses were performed using a 12-bit CCD digital camera equipped with a Moritex high-definition lens (ML-U3514MP9, Jingzhou, China) and analyzed using Vic-2D software. Test data were recorded at a camera frame rate of 15 Hz and synchronized with the loading system measurements. A fine, randomly distributed speckle pattern was generated on the specimen surface using black spray paint to create sufficient contrast and a random speckle pattern, ensuring optimal contrast for the DIC post-processing algorithm.

## 3. Results and Discussion

**Figure 2:**
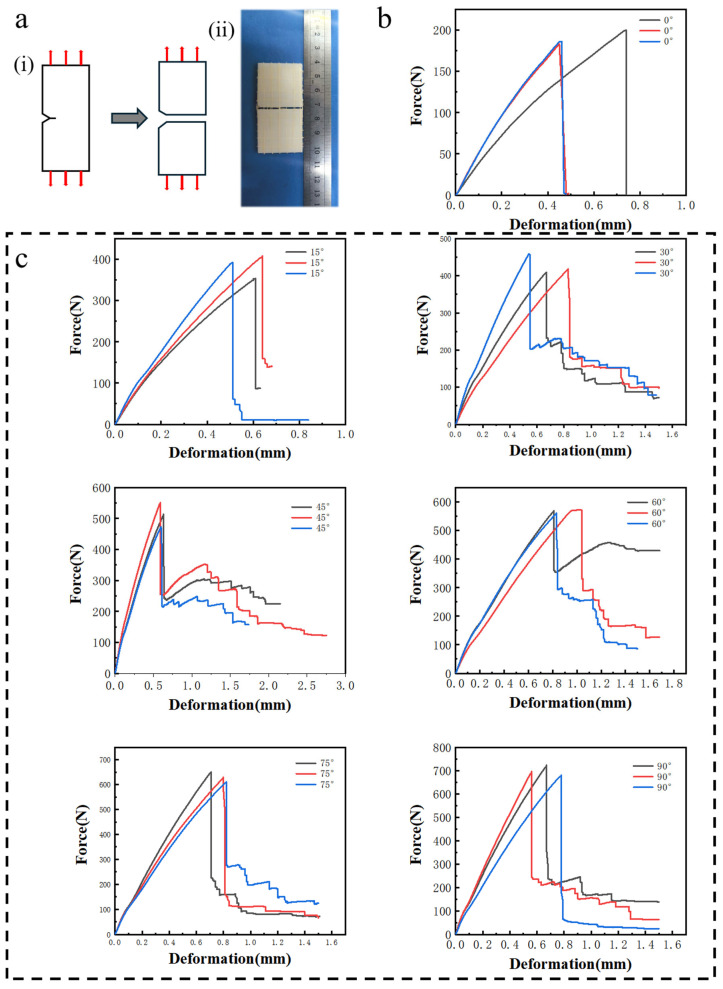
Mechanical performance of UV-curable standard resin before and after the addition of flax fibers. (**a**) Fracture mode of the printed samples (i) and representative sample after fracture with an interlayer angle of 90° (ii). (**b**) Force–displacement curve of the UV-curable standard resin. (**c**) Force–displacement curves of the UV-curable standard resin with different flax fiber interlayer angles (15°, 30°, 45°, 60°, 75°, 90°). The samples for tests were repeated three times.

Figure 2a(i) shows the force–displacement curves of samples with different interlayer angles at a loading rate of 1 mm/min. In addition, Figure 2a(ii) shows a representative post-fracture image of a sample after fracture with an interlayer angle of 90°. Table 2 lists the maximum force, fracture work, tensile strength, and fracture energy. Figure 2b shows the tensile behavior for a UV-curable standard resin printed structure, characterized by a brittle failure mode with a maximum force of 191.64 N. Figure 2c shows that a pronounced enhancement in the force–displacement curve occurs after the samples were reinforced with flax fibers. The maximum force of samples with different interlayer angles increased progressively with the rotation angle. The results indicate that the fiber-reinforced UV-curable standard resin exhibited the highest failure force at an interlayer angle of 90°. The composite exhibited a work of fracture 5.94 times higher than the unreinforced resin, demonstrating superior strength and toughness.

**Figure 3:**
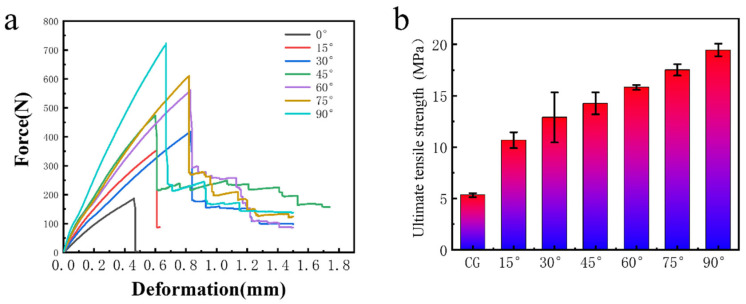
Tensile test of the samples with flax fiber interlayer angles of CG (control group), 15°, 30°, 45°, 60°, 75°, and 90°. (**a**) Force–displacement curves. (**b**) Ultimate tensile strength.

**Figure 4:**
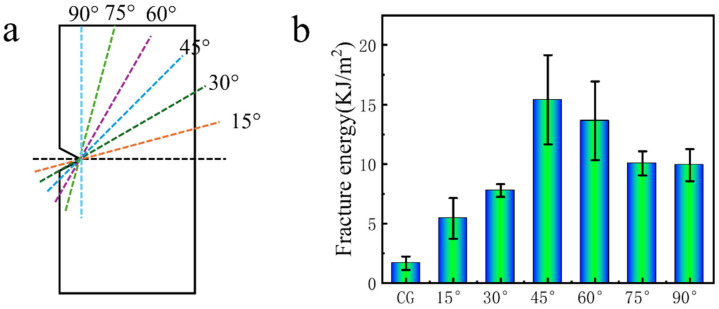
(**a**) Schematic diagram of flax fiber interlayer angles. (**b**) Fracture energy of the samples with flax fiber interlayer angle of CG (control group), 15°, 30°, 45°, 60°, 75°, and 90°.

Figure 3 illustrates the relationship between mechanical performance and the inter-layer angle of flax fibers. Figure 3a shows the force–displacement curves of seven representative specimens, including the Control Group (CG) without fiber rotation and composites with interlayer angles of 15°, 30°, 45°, 60°, 75°, and 90°. A clear improvement in the peak force at failure can be observed with increasing interlayer angle. The corresponding ultimate tensile strengths are summarized in Figure 3b, revealing a monotonic increase in tensile strength as the interlayer angle increases from 0° (CG) to 90°. Quantitatively, the CG sample exhibits an ultimate tensile strength of 5.32 ± 0.19 MPa, serving as the baseline for evaluating the reinforcement effect. Introducing a small interlayer rotation of 15° nearly doubles the tensile strength to 10.68 MPa, representing an increase of approximately 101% relative to the CG. As the interlayer angle increases to 30°, the ultimate tensile strength further rises to 12.89 MPa, corresponding to a 142% increase compared to the CG and a 20.7% improvement over the 15° configuration. At an interlayer angle of 45°, the composite reaches an ultimate tensile strength of 14.25 MPa, which is 168% higher than that of the CG. This configuration marks a critical transition point where the fibers are optimally oriented to resist both tensile and shear stresses, resulting in effective load transfer across layers and enhanced crack deflection. Increasing the interlayer angle to 60° yields a further improvement in tensile strength to 15.81 MPa, corresponding to a 197% increase relative to the CG. However, the incremental gain from 45° to 60° (approximately 11.0%) becomes more moderate, suggesting a gradual saturation of reinforcement efficiency. When the interlayer angle reaches 75°, the ultimate tensile strength increases to 17.51 MPa, achieving a 229% enhancement over the CG. The highest tensile strength is observed for the 90° sample, which reaches 19.45 ± 0.61 MPa with an increase of approximately 266% compared to the CG. This improvement indicates the dominant role of fiber orientation in governing tensile performance. At higher interlayer angles, the helicoidal architecture promotes more uniform stress distribution, suppresses strain localization, and enables higher energy absorption prior to failure. These results demonstrate that increasing the interlayer rotation angle of flax fibers leads to a significant enhancement in tensile strength. Importantly, while higher interlayer angles maximize tensile strength, the optimal balance between strength, toughness, and crack-deflection capability occurs at intermediate angles, particularly at 45°, which is shown in Figure 4.

Fracture energy represents the energy absorption capacity of a material during its fracture process. Figure 4a illustrates a schematic of the helicoidal alignment of flax fiber at various interlayer angles. Figure 4b depicts the fracture energy results for different interlayer angles, revealing a trend of an initial increase followed by a subsequent decrease in energy absorption. Fracture energy represents the energy required to propagate a unit area of crack, reflecting intrinsic material toughness, whereas work of fracture represents the total energy absorbed by the specimen, calculated from the area under the force–displacement curve. For the fiber-free UV-cured standard resin (control group), the measured fracture energy was 1.67 ± 0.19 KJ/m^2^, and the fracture work was 60.10 J. Both values increased after embedding flax fibers at different interlayer angles. For example, at an interlayer angle of 45°, the fracture energy reached 15.41 ± 1.07 KJ/m^2^, an increase of approximately 823% compared to the control group, while the fracture work increased to 554.74 J. These results indicate that the addition of flax fibers significantly enhances the toughness of the UV-curable standard resin. We conducted a comparison across all interlayer angles to show the evolution of energy dissipation mechanisms in the helicoidal flax fiber–reinforced composites in details. As summarized in Figure 4b and Table 2, the control group (CG) without fiber reinforcement exhibits a low fracture energy and work of fracture, indicating the intrinsically brittle nature and limited energy absorption capability of the UV-cured standard resin. Upon introducing flax fibers with a small interlayer rotation of 15°, the fracture energy increases markedly to 5.43 KJ/m^2^, corresponding to an absolute increase of 3.76 KJ/m^2^ and a relative enhancement of approximately 225% compared to the CG. Meanwhile, the work of fracture rises to 129.70 J, more than doubling that of the control sample. This improvement indicates that even a modest helicoidal arrangement is sufficient to obtain additional toughening mechanisms. As the interlayer angle increases to 30°, the fracture energy further increases to 7.78 KJ/m^2^, representing a 366% enhancement relative to the CG, while the work of fracture reaches 279.96 J. Compared with the 15° configuration, the fracture energy increases by 43.3%, and the fracture work increases by approximately 116%, indicating a pronounced enhancement in energy absorption during crack propagation. At this stage, the rotated fiber architecture promotes crack–fiber interactions. The most significant enhancement is observed at an interlayer angle of 45°, where the fracture energy reaches a maximum value of 15.41 KJ/m^2^ with an increase of approximately 823% compared to the control group. Correspondingly, the work of fracture increases to 554.74 J, which is more than 9 times higher than that of the CG. This improvement indicates the critical role of the 45° helicoidal configuration in maximizing fracture energy and damage tolerance of the composite. At this optimal angle, fibers are oriented to resist combined tensile and shear stresses, enabling extensive crack deflection, fiber pull-out, and interfacial debonding. These mechanisms enlarge the fracture process zone and promote stable crack growth, thereby allowing the 3D printed composite to absorb substantially more energy prior to failure.

Beyond 45°, however, a gradual decline in the fracture energy and the fracture work is observed. At an interlayer angle of 60°, the fracture energy decreases to 13.64 KJ/m^2^, while the work of fracture drops to 491.04 J. Although these values remain higher than those of the CG, the reduction compared to the 45° sample suggests a diminishing contribution of toughening mechanisms. Further increasing the interlayer angle to 75° and 90° leads to additional decreases in fracture energy, reaching 10.06 KJ/m^2^ and 9.91 KJ/m^2^, respectively, with corresponding fracture work values of 361.98 J and 356.79 J. At these higher angles, fibers tend to align more perpendicular to the primary crack propagation direction, which limits interlayer shear transfer and reduces the effectiveness of crack deflection and energy dissipation through fiber rotation and sliding. These results show that a non-monotonic dependence of fracture energy on interlayer angle, characterized by an initial increase followed by a decrease at higher angles. The existence of a clear maximum at 45° indicates an optimal balance between the fracture energy and damage tolerance, rather than solely optimizing tensile strength.

**Figure 5:**
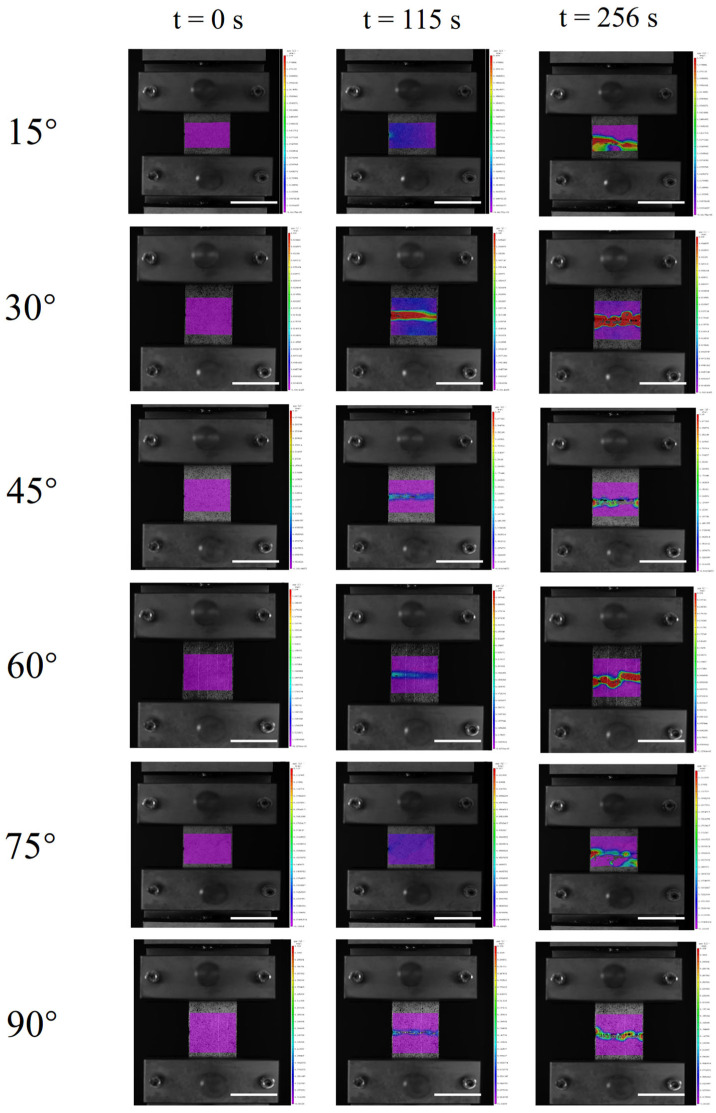
DIC measurements of *ε_yy_* of the samples with flax fiber interlayer angles of 15°, 30°, 45°, 60°, 75°, and 90°. The scale bars are 3 cm.

To further investigate the underlying toughening mechanisms, representative specimens incorporating bio-inspired architectures were systematically examined using the DIC method, as presented in Figure 5. The DIC measurements were conducted on the same specimens subjected to the fracture toughness tests to ensure a direct connection between deformation behavior and fracture response. Prior to testing, the specimen surfaces were coated with a thin white paint layer, followed by the application of a random black speckle pattern to provide sufficient grayscale contrast required for displacement tracking. During uniaxial tensile loading, a synchronized camera system continuously recorded the deformation evolution of the specimens. The acquired image sequences were subsequently analyzed to extract full-field displacement and strain maps. In particular, the normal strain component in the loading direction (*ε_yy_*) was analyzed at representative time points of t = 0, 115, and 256 s to capture the progression of strain localization and redistribution during crack initiation and propagation. As shown in Figure 5, at the initial loading stage, strain localization was confined to a narrow region near the pre-crack tip, indicating deformation dominated by stress concentration. As the fracture propagated toward the flax fiber, strain became distributed at multiple locations, forming a larger process zone. The increasing interlayer angle led to higher tensile strain. Failure initiated at the pre-crack location and progressed along the pre-machined crack path under tensile loading. Based on the analysis of crack propagation patterns, cracks tended to propagate through relatively weaker interfaces.

**Figure 6:**
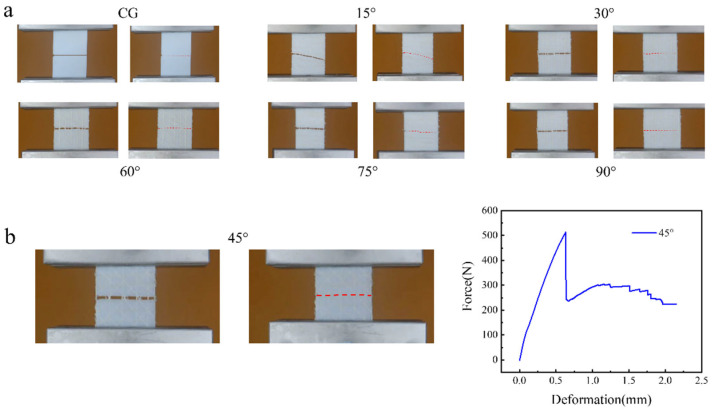
Crack propagation patterns of specimens with flax fiber incorporation at different interlayer angles. (**a**) CG, 15°, 30°, 60°, 75°, 90°. (**b**) Crack propagation path and force–displacement curve at 45°.

Figure 6a presents the crack propagation patterns of specimens with flax fiber structures at varying interlayer angles. In the CG group, cracks propagated directly along the pre-existing crack line. Samples with a 45° interlayer angle exhibited a fracture pattern where, after initial cracking, the flax fibers remained intact radially, and the crack extended along a quasi-linear path, penetrating through both layers of flax fibers and resin, demonstrating improved crack-resisting capability. For samples with other angles (15°, 30°, 60°, 75°, 90°), cracks propagated along irregular paths due to the varying orientations of the flax fibers. Increasing the interlayer angle from 15° to 45° resulted in increased fracture toughness, while increasing it from 45° to 90° led to a reduction in fracture toughness. Furthermore, the initial crack propagation was consistent with the patterns identified through DIC analysis. Figure 6b displays the fracture pattern and the force–displacement curve for the sample with a 45° interlayer angle. The reinforcement effect of flax fibers arranged at 45° angles results in a multi-stage fracture process, which enhances the mechanical performance (particularly fracture toughness) of the composite. The angled arrangement improves stress distribution, facilitates crack deflection and branching, and promotes energy dissipation across multiple fractures. Thus, the sample exhibits progressive fracture behavior. These properties are crucial for structural applications where high resistance to mechanical stress and impact loading is essential.

## 4. Conclusions

In conclusion, this work explored the toughening effects of incorporating flax fibers at various interlayer angles into UV-curable standard resin. Inspired by the Bouligand structure, a natural helicoidal architecture, we designed layered composites using photopolymerization-based 3D printing, where flax fibers were incrementally rotated between layers to reproduce this biomimetic pattern. The findings show distinct anisotropic fracture behavior in printed specimens. Among the interlayer angles evaluated, the Bouligand-inspired architecture 45° sample exhibited the highest resistance to cracking, followed by the 60° sample. In the 45° samples, cracks propagated through both the printed layers and the interfacial regions. The inclusion of flax fiber with a 45° interlayer angle enhanced shear resistance and redirected stress along multiple pathways, effectively reducing stress concentrations, thereby markedly enhancing fracture toughness. In contrast, although the 60° sample also benefited from the helicoidal arrangement, its maximum shear stress was lower than that of the 45° sample, leading to reduced fracture-toughening effectiveness. Therefore, incorporating flax fibers improves the fracture toughness of standard resin, with the degree of enhancement highly dependent on the interlayer angle of incorporation. Notably, the 45° interlayer angle offers the optimal balance between structural reinforcement and crack-deflection capability, delivering the most pronounced improvement in fracture performance.

Our work also demonstrates that the Bouligand-inspired flax fiber–reinforced composites offer promising opportunities for lightweight, damage-tolerant, and sustainable material systems in applications such as impact-resistant components, soft robotics, and architected structural materials enabled by UV photopolymerization 3D printing. Several limitations of the present work should be acknowledged. The number of printed layers and the total structural thickness were relatively limited, which may restrict the full development of helicoidal toughening mechanisms observed in natural Bouligand architectures. Future studies could therefore explore increased layer numbers and finer interlayer rotation increments to further improve crack deflection and energy dissipation. In addition, only a single UV-curable resin system and one type of natural fiber were investigated. In future works, developing multiple resin formulations, hybrid or functional fillers, and multi-material printing strategies would enable broader mechanical tunability and multifunctionality of 3D printed high-performance bio-inspired composites.

## Figures and Tables

**Table 1:** Samples with different flax fiber orientations.

Samples	Matrix	Fiber	Fiber Orientation
Sample S1	Standard Resin	N/A	N/A
Sample S2	Standard Resin	Flax fiber	15°
Sample S3	Standard Resin	Flax fiber	30°
Sample S4	Standard Resin	Flax fiber	45°
Sample S5	Standard Resin	Flax fiber	60°
Sample S6	Standard Resin	Flax fiber	75°
Sample S7	Standard Resin	Flax fiber	90°

**Table 2:** Performance comparison between different layer angles.

	Control Group (CG)	15°	30°	45°	60°	75°	90°
Max Force (N)	191.64	384.48	464.09	513.13	569.14	630.26	700.22
Fracture energy (KJ/m^2^)	1.67	5.43	7.78	15.41	13.64	10.06	9.91
Work of fracture (J)	60.10	129.70	279.96	554.74	491.04	361.98	356.79
Ultimate tensile strength (MPa)	5.32	10.68	12.89	14.25	15.81	17.51	19.45

## Data Availability

Data will be made available on reasonable request.
